# Can We Improve Structured Sequence Processing? Exploring the Direct and Indirect Effects of Computerized Training Using a Mediational Model

**DOI:** 10.1371/journal.pone.0127148

**Published:** 2015-05-06

**Authors:** Gretchen N. L. Smith, Christopher M. Conway, Althea Bauernschmidt, David B. Pisoni

**Affiliations:** 1 Department of Psychology, Georgia State University, Atlanta, GA, United States of America; 2 Department of Psychology, St. Bonaventure University, St. Bonaventure, NY, United States of America; 3 Department of Psychological and Brain Sciences, Indiana University, Bloomington, IN, United States of America; UNLV, UNITED STATES

## Abstract

Recent research suggests that language acquisition may rely on domain-general learning abilities, such as structured sequence processing, which is the ability to extract, encode, and represent structured patterns in a temporal sequence. If structured sequence processing supports language, then it may be possible to improve language function by enhancing this foundational learning ability. The goal of the present study was to use a novel computerized training task as a means to better understand the relationship between structured sequence processing and language function. Participants first were assessed on pre-training tasks to provide baseline behavioral measures of structured sequence processing and language abilities. Participants were then quasi-randomly assigned to either a treatment group involving adaptive structured visuospatial sequence training, a treatment group involving adaptive non-structured visuospatial sequence training, or a control group. Following four days of sequence training, all participants were assessed with the same pre-training measures. Overall comparison of the post-training means revealed no group differences. However, in order to examine the potential relations between sequence training, structured sequence processing, and language ability, we used a mediation analysis that showed two competing effects. In the indirect effect, adaptive sequence training with structural regularities had a positive impact on structured sequence processing performance, which in turn had a positive impact on language processing. This finding not only identifies a potential novel intervention to treat language impairments but also may be the first demonstration that structured sequence processing can be improved and that this, in turn, has an impact on language processing. However, in the direct effect, adaptive sequence training with structural regularities had a direct negative impact on language processing. This unexpected finding suggests that adaptive training with structural regularities might potentially interfere with language processing. Taken together, these findings underscore the importance of pursuing designs that promote a better understanding of the mechanisms underlying training-related changes, so that regimens can be developed that help reduce these types of negative effects while simultaneously maximizing the benefits to outcome measures of interest.

## Introduction

The acquisition of natural language may be one of the more formidable tasks facing human beings, and yet we are exceptionally proficient at it. Understanding the core mechanisms responsible for the ability to learn language has been a particular challenge in cognitive neuroscience. One long-standing, prominent argument has been that humans have dedicated domain-specific neural mechanisms that evolved specifically for language acquisition [1]. An alternative hypothesis is that language acquisition may rely heavily on domain-general learning and processing abilities that allow the learner to utilize the structure inherent in language [2], [3]. One such domain-general mechanism that may support language acquisition has been referred to as structured sequence processing (SSP), or the ability to extract, encode, and represent structured patterns occurring in a temporal sequence [4], [5], [6]. If SSP supports language, then it may be possible to improve language function by enhancing this basic learning ability, similar to the way that computerized working memory (WM) training has been demonstrated to transfer to non-trained tasks of executive function, attention, and other aspects of cognition [7], [8], [9], [10], [11]. The possibility of improving SSP through adaptive training, with improvements generalizing to aspects of language processing, has both clinical and theoretical ramifications as it would not only identify a novel intervention to treat language impairments but would also identify a more direct relationship between SSP and language.

In this paper, we will first review recent evidence that SSP may underlie language learning, underscoring the scarcity of literature using experimental manipulations to investigate the two processes. We will then review relevant findings from the WM training literature, which served as inspiration for the structure-based sequence training regimen used in the present study. This background will then lead to a description of our study using a novel computerized sequence training task and a mediational analysis to explore the relationship between SSP and language function.

### Previous Evidence Linking SSP to Language

SSP, sometimes referred to as sequential learning or statistical learning, allows people to learn about structured patterns of information in the environment in a relatively automatic and unconscious fashion [12], [13], [6]. Importantly, SSP abilities may be especially crucial for the development of social and linguistic knowledge [14], [5]. SSP allows the language learner to detect and to utilize the structure inherent in phonology [13], syntax [15], and word order [5]. Furthermore, impairments in SSP may contribute to a number of communication disorders, including dyslexia [16], specific language impairment [17], and language delays observed in hearing impairment [18].

Research findings have indicated that individual differences in performance on non-linguistic sequential learning tasks are significantly correlated with how healthy typically-developing adults perform on a degraded speech perception task, in which participants must use preceding context to predict upcoming units of speech [5], [19], see also [20]. Furthermore, Christiansen et al. [21] provided a within-subject comparison of the neural mechanisms supporting visual sequence learning and language processing using event-related potentials (ERPs). The key finding was that sequences containing structural irregularities in the SSP task elicited a P600-like component that was statistically identical to the P600 elicited by syntactic violations in the natural language task. This outcome suggests that the same neural mechanisms may be recruited for both SSP and language processing, a finding that is also substantiated by other neuroimaging studies showing that Broca’s area is active in both language and non-language SSP tasks e.g., [22], [23]; for reviews, see [24], [6].

Although these studies are highly promising, more direct evidence of a link between SSP and language is needed. One particularly striking void in the type of approach used thus far is the pursuit of designs that incorporate experimental manipulations designed to improve SSP, as a way to help demonstrate an underlying link between SSP and language. Even a first step in this direction could have potential implications for how to improve language functioning in typical and atypical development.

### Cognitive Training

If language development relies, at least in part, on SSP, then it may be beneficial to improve language function by enhancing SSP itself. One strategy for this might be to stimulate neural regions, such as Broca’s area, that are thought to underlie SSP using magnetic or electrical stimulation e.g., [25]. Another possibility would be to use computerized neurocognitive training techniques to improve SSP. One cognitive domain that has received much interest in the cognitive training literature and may be relevant to the current endeavor is WM. WM refers to the temporary storage and manipulation of information necessary for complex cognitive tasks [26]. While the training tasks and populations have varied, the general goal of a number of recent studies has been to determine whether WM training tasks could improve WM capacity and show transfer to non-trained tasks of spatial and verbal WM, attention, and other cognitive functions. Sometimes this distinction is referred to as “near transfer” (transfer effects to constructs closely related to the training technique itself) and “far transfer” (transfer effects to other constructs thought to have a theoretical link with what is being trained). For example, Klingberg et al. [7], [8] used a WM training task (Cogmed Systems, Stockholm, Sweden) with children with ADHD. The children performed visuospatial and/or verbal WM tasks over a period of 5 weeks. The visuospatial task involved remembering the position of objects on a 4 x 4 grid, and the verbal task involved remembering phonemes, letters or digits [8]. Importantly, difficulty level of the tasks was adjusted to match each child’s WM ability by changing the number of elements that had to be recalled. A control group performed the same tasks except the length of items to recall stayed fixed rather than being adaptively adjusted. At post training sessions both 5 to 6 weeks and 3 months following the pre measures, children showed significant improvement to performance on WM and executive functions (as measured by a visuospatial Span board task, digit span, Stroop task, and Raven’s matrices) compared to a control group.

Overall, the results from a number of recent WM training studies suggest that WM can be improved through adaptive training [27], [28], [29]. Furthermore, training on an adaptive visuospatial task can also transfer in a domain-general manner to nontrained tasks of WM (demonstrating near transfer) but also to other cognitive functions such as inhibition [7], [8], [9], attention [11], and verbal WM [10] (demonstrating far transfer).

Although there have been some questions about the effectiveness of WM training [30], [31], and especially in relation to the far transfer results, these findings raise the possibility that WM and other aspects of cognition, perhaps even SSP, can be modifiable by intensive training. The difference between the standard WM task and SSP is that WM involves recalling a series of stimuli that have no structural relationship to each other; that is, the stimuli are presented in a random order. On the other hand, many of our interactions with the world involve encoding, storing, and processing structural regularities, in which SSP is recruited. For this reason, we propose that to target and train SSP it is necessary to embed structured rather than random sequences into the training protocol.

To summarize, the previous evidence suggests that language functions may rely, at least in part, on SSP. Furthermore, recent findings from the WM training literature are promising because they offer the possibility that SSP could be improved, which if successful, could possibly lead to improvements to language processing. Finally, it may be beneficial to use the results from this type of intervention design in conjunction with process modeling techniques to provide more insight about the putative underlying relations among the constructs. We will now describe the present study, which combines all of these elements to specifically test whether adaptive training with structural regularities has a contributory impact on language via the mediation of SSP.

### The Present Study

The goal of the present study was to investigate the putative underlying relationship between domain-general structure-based learning mechanisms (i.e., SSP) and language functions by exploring the effect of adaptive sequence training with structural regularities (SSP training) versus adaptive sequence training without structural regularities (WM training), relative to a control or reference group. Participants in the SSP training group engaged in a 4-day computerized sequence training regimen that involved viewing and then reproducing visual-spatial structured sequences. This type of training is similar to previous WM training studies; however, our novel manipulation for this group was the introduction of sequences containing structural regularities. Thus, rather than having those participants trained with random sequences (as in the case of all previous WM training studies), they were trained with non-random, structured patterns. For the second group, which we refer to as the WM training group, these participants engaged in the same computerized sequence training task but instead of using structured sequences, the sequences contained no structure, consistent with all other previous WM training studies.

Participants received a battery of cognitive tests on the first day, then four days of either the SSP training condition, the WM training condition, or the control condition (which, similar to the control groups used in previous WM training studies, consisted of a non-adaptive computerized training that is not expected to result in cognitive improvements). On the sixth day participants received the same battery of cognitive tests that were administered on the first day in order to measure changes to non-trained tasks. The group of participants engaging in the adaptive sequence training with structural regularities (SSP training, Group 1) and the group of participants engaging in the adaptive sequence training without structural regularities (WM training, Group 2) were both compared to a control group that engaged in nonadaptive, unstructured sequences (Group 3).

1. Thus, the first hypothesis for this study was simply that only the SSP training would result in improvements to both a non-trained SSP task and a language processing task. This hypothesis was examined through the use of multivariate analysis of variance (MANOVA) comparing group means of SSP and language processing.

However, regardless as to the results of the MANOVA, there is a second and perhaps more useful way to analyze these data. Whereas all of the previous WM training studies used as their primary analyses a comparison of the means on non-trained task from pre- to post-training, the present study also used a mediation model. This analysis approach has the ability to explore the core mechanisms or processes underlying any such relation between cognitive training and language processing by investigating whether there exists a mediating relationship among the variables. It can also help tease apart the separate influences relating to near and far transfer effects, and help explain why one or the other might be present. With the mediation model technique, we may be better able to understand the effects of cognitive training on SSP and language processing. A MANOVA or other regression technique does not allow for the identification of mediating variables and, thus, is unable to help clarify the nature of the relations among training, SSP, and language processing.

A mediation analysis allows one to explain the mechanism by which one variable influences, or has an effect, on another [32], [33]. Both “direct” and “indirect” effects can be tested [32], [33]. Direct effects are when the independent variable (IV) directly impacts the dependent variable (DV). Indirect effects are when the IV impacts the DV through the mediation of a third variable, called the mediator (M). Hayes and Preacher [33] introduced a technique for conducting a mediation analysis when the IV is multicategorical, as in the case of the present study comparing two experimental groups to a control group. Since the effects of being in one of the experimental groups are in comparison to the effects of being in a reference group, they suggest using the terms “relative direct effects” and “relative indirect effects” [33]. In the present study, the IV is training type [i.e., whether participants receive SSP training (Group 1), or WM training (Group 2), with both compared to the control participants (Group 3]. The DV is language processing (measured at post-training). The M is SSP ability (also measured at post-training). Thus, our aim is to test the relative direct and indirect effects through which cognitive training might affect language processing. Specifically, does cognitive training have a relative direct impact on language processing? And, does cognitive training have a relative indirect effect on language processing by impacting SSP ability?

**Fig 1:**
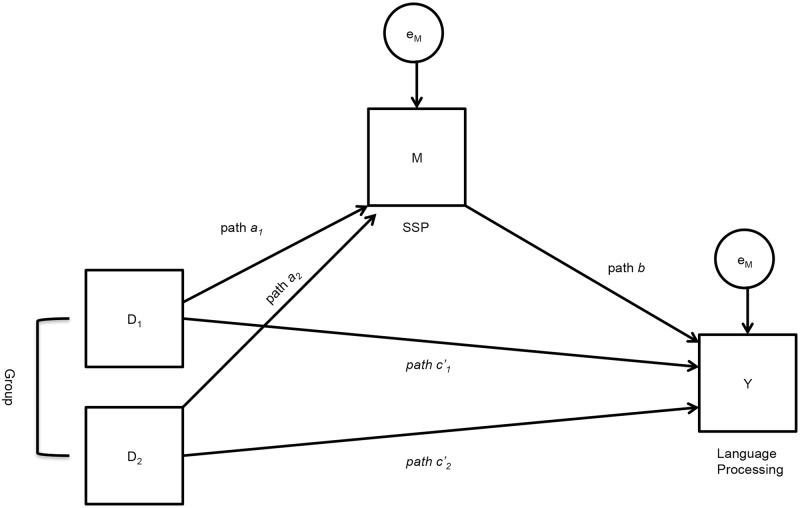
Untested mediation model. The untested model predicting a significant indirect effect (patch *a*_1_ x *b*) suggesting that adaptive sequence training with structural regularities would have a significant overall positive impact on language processing by way of the mediating variable, SSP. It also predicts a significant direct effect (path *c'*_1_), suggesting adaptive sequence training with structural regularities would have a positive impact directly on language processing.

The untested model is presented in Fig 1. For both experimental training groups (i.e., Group 1 and Group 2), path *a* x *b* represents the relative indirect effect of the IV (training type) on the DV (language) via the mediator, M (SSP) (relative to the reference group, Group 3). Path *a* represents the effect of the IV on M, whereas path *b* represents the effect of M on the DV. On the other hand, path *c'* represents the relative direct effect of the IV on the DV (relative to the reference group, Group 3). The relative total effect (path *c*, not depicted in the untested model) would represent the sum of the direct and indirect effects (compared to the reference group). Again, our focus was testing, for Group 1 and Group 2, the relative indirect and relative direct effects, represented by path *a* x *b* and path *c'*, respectively. Additionally for both groups, we tested the individual components of the relative indirect effect, represented by path *a* and path *b*. We did not make an *a priori* hypothesis about the relative total effect for both groups, represented by path *c*.

Thus, in association with the mediation model, we assessed the following hypotheses:

1. We predicted that there would be a significant relative indirect effect (path *a x b*) only for the SSP training group (Group 1). That is, we predicted that adaptive sequence training with structural regularities would have an underlying impact on language processing by way of the mediating variable, SSP. In terms of the individual components of this indirect path, we predicted a significant (positive) effect of path *a*; that is, adaptive sequence training with structural regularities would have a significant positive impact on SSP. We also predicted a significant (positive) effect of path *b*; that is, SSP would have a significant positive impact on language processing.
2. We also predicted that there would be a significant relative direct effect (path *c'*) only for the SSP group (Group 1). That is, we predicted that adaptive sequence training with structural regularities would have a positive effect overall on language processing.

## Method

### Ethics Statement

The study was approved by the Indiana University Institutional Review Board. Participants provided written consent prior to participation.

### Participants

Sixty-six participants (38 males and 28 females, ages 18–30) were recruited at Indiana University to participate in this study for monetary compensation. All participants were native speakers of English and reported no history of a hearing loss, speech impairment, or other cognitive/perceptual/motor impairments at the time of testing.

### Materials

All visual stimuli/sequences were presented using a *Magic Touch* touch-sensitive monitor and a Macintosh Power PC G4. Responses were made on the *Magic Touch* touch-sensitive monitor. For the measure of language, the auditory stimuli were presented using Beyer dynamic DT100 headphones. All experimental tasks for this experiment took place in a sound-attenuated booth (Industrial Acoustics Company).

### Procedure

Participants took part in the experiment for 6 days, with no more than 2 intervening days between sessions. On Day 1 they were assessed on several measures, two of which we focus on in the present manuscript: a measure of SSP and a measure of language (speech recognition in noise). During the next 4 days participants received either adaptive sequence training with structural regularities (Group 1, SSP training), adaptive sequence training without structural regularities (Group 2, WM training), or the control condition of non-adaptive unstructured sequences (Group 3, reference group)). On Day 6 participants were re-assessed on the same measures as Day 1. An overview of the study design is given in Table 1.

**Table 1:** Overview of study design.

Day 1 Pre-Training	Days 2–5 Sequence Training	Day 6 Post-Training
Speech Recognition In Noise	Group 1 Adaptive, Structural Regularities	Speech Recognition In Noise
Statistical-Sequential Learning	Group 2 Adaptive, No Regularities	Statistical-Sequential Learning
	Group 3 Non-adaptive, No Regularities	

#### Pre-training measures

Although several other pre-training measures were administered as part of a separate study reported in Conway et al. [5], in the present analysis we focus on only the measures of SSP and language. Participants in each of the three groups completed the measure of SSP first, the measure of language second, and the other cognitive assessments last. Below we briefly describe each measure; further details are provided in Conway et al. [5].

#### Measure of structured sequence processing

The measure of SSP was identical to the “Simon” visual statistical-sequential learning task used in previously published work, see [5], [34] for full details. Performance on this task has been shown to be significantly correlated with language processing skills in healthy adults [5] and in language-delayed children [18]. In this task, participants viewed a series of 4 colored squares that light up on the touchscreen and were asked to reproduce each sequence they had just observed by touching the appropriate color.

The task consisted of two phases, a learning phase and a test phase. In both phases, the participant’s task was the same: to reproduce each sequence immediately following presentation by touching the colored squares displayed on the touch-sensitive monitor in the correct order. No feedback was given. However, unbeknownst to participants, the phases differed in terms of the types of temporal sequences that were presented. In the learning phase, the sequences were not random but rather were generated according to an underlying artificial grammar that specified the probability of a particular element in a sequence occurring given the preceding element (see Table 2). In the learning phase, 48 sequences generated from the grammar were presented once each, in random order. In the test phase, 40 new sequences were presented: 20 sequences generated from the artificial grammar and 20 sequences that were pseudo-random (i.e., the occurrence of each element in the sequence was random except that no element could follow itself). The participants were not told that this was a test phase or that there were different types of sequences used in the experiment. From the perspective of the participant, the test phase was just the same sequence reproduction task they had been doing as before.

**Table 2:** Artificial grammars used to generate the order of stimuli.

	Constrained Grammar (n+1)				Unconstrained Grammar (n+1)			
Colors/locations (n)	1	2	3	4	1	2	3	4
1	0.0	0.5	0.5	0.0	0.0	0.33	0.33	0.33
2	0.0	0.0	1.0	0.0	0.33	0.0	0.33	0.33
3	0.5	0.0	0.0	0.5	0.33	0.33	0.0	0.33
4	1.0	0.0	0.0	0.0	0.33	0.33	0.33	0.0

#### Scoring

In the test phase, a sequence was scored as correct if a participant correctly reproduced it. A score was given for each correctly reproduced sequence based on its length (i.e., a correctly reproduced sequence of length 5 was given a score of 5). As in previous studies [5], [18], [34] a learning score was then obtained by subtracting the total score for the ungrammatical sequences in the test phase from the grammatical sequences in the test phase. A higher learning score indicates better performance on novel statistically-structured sequences compared to random ones, suggesting that successful statistical-sequential learning has occurred.

#### Measure of language

The measure of language was a speech recognition in noise task that required participants to listen to 50 spectrally degraded sentences and then to write down the last word that they heard in each sentence, see [5] for full details. The sentences varied in terms of the final word’s predictability: high-predictability sentences (N = 25) had a final word that was highly predictable given the sentence context (e.g., “*Greet the heroes with loud* *cheers*”); whereas anomalous sentences (N = 25) had a final word that was not predictable given the sentence context (e.g., “*The burglar was parked by an* *ox*”). Sentences were presented in random order using a self-paced format.

#### Scoring

Responses were scored based on the number of correct final words for each of the two sentence types (high-predictability and anomalous). As in Conway et al. [5], a difference score was computed as the number of final words identified in high-predictability sentences minus the number of final words in anomalous sentences. This difference score reflects how well a participant is able to use sentence context to help perceive the final word in the sentence, a form of top-down language processing that has been argued to depend on how well one is able to track statistics in language [5]. For this reason, we expected that any improvements to SSP might carry over to improvements in how well participants are able to use sentence context to perceive words under poor listening conditions.

#### Sequence training task

**Fig 2:**
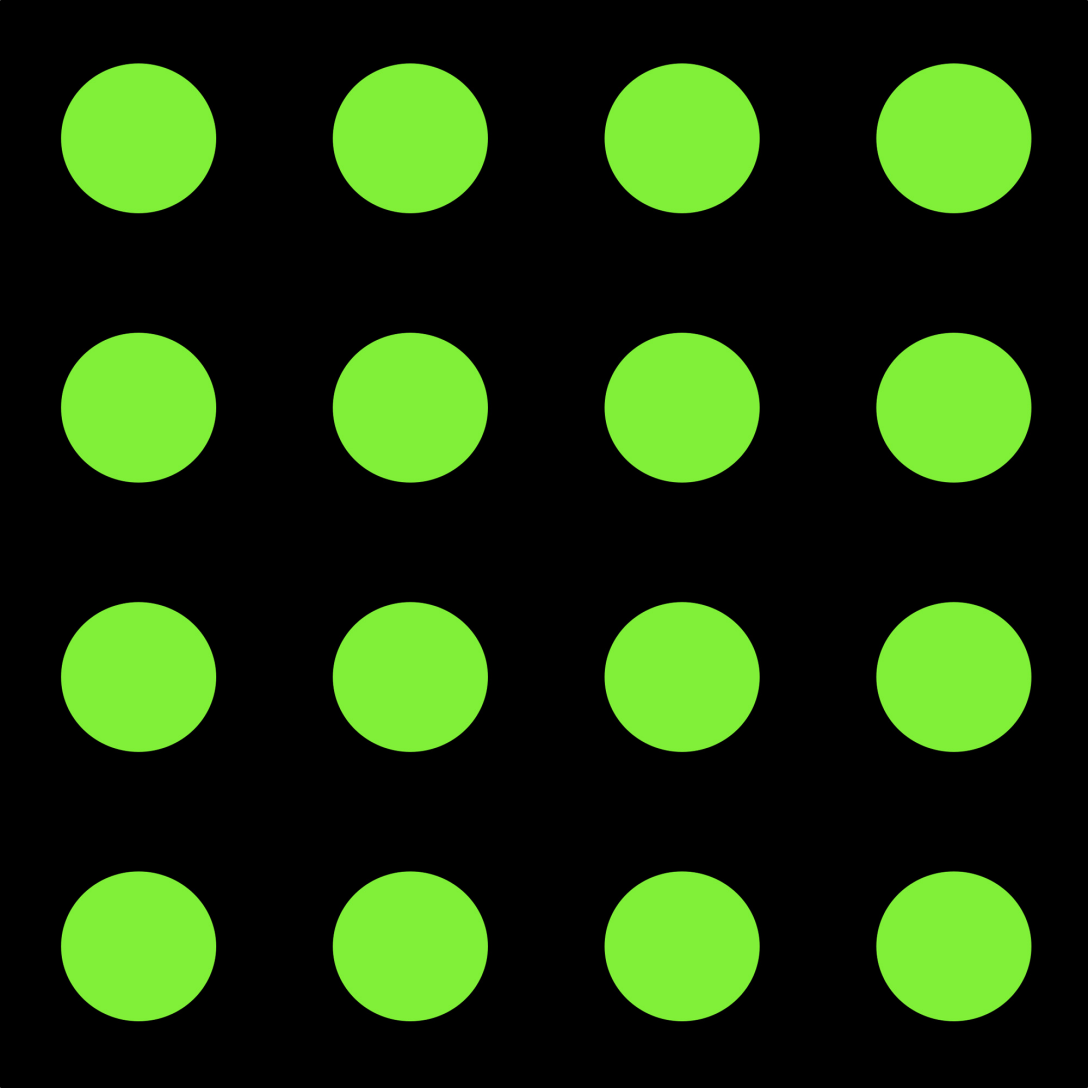
Training task. Touch screen of 16 circles arranged in a 4 x 4 display for visuospatial sequence training task.

The training task began in a separate session (on a different day) after the completion of the pre-training assessments. The sequence training task consisted of several blocks of sequence-reproduction trials that either adaptively increased in length as participants’ performance improved (Groups 1 and 2) or were non-adaptive (Group 3). On each trial, participants saw a series of green circles light up on the touchscreen monitor and were asked to reproduce the sequence they had just seen (see Fig 2). Sixteen circles were arranged on a 4x4 grid, inspired by the type of computerized training programs used by Klingberg et al. [7], [8]. Individual circles on the 4x4 grid were illuminated for 250ms and were off for 250ms between elements in the sequence. The participants’ task was to reproduce each sequence immediately following presentation by touching the green circles displayed on the touch-sensitive monitor in the correct order. As participants made their responses, each circle they pressed stayed illuminated for 250ms. At the end of the presentation of a sequence if the participant did not make a response within 2 seconds a new sequence was presented. Each session of the training task consisted of 3 blocks of 50 trials.

The order that the circles lit up either was dependent upon embedded structural regularities (Group 1) or was pseudorandom and did not follow structural regularities (Groups 2 and 3). The sequences with underlying structural regularities were generated so that each element in the sequence could be followed by 3 others in the set with equal likelihood. The sequences without underlying structural regularities were generated so that each element in the sequence could be followed by any other in the set with equal likelihood (that is, any of the 15 other elements). The underlying sequences themselves were the same for all participants (within each group). However, the actual mapping between each sequence element and each circle on the screen was randomly determined for each participant. Thus, while the underlying sequences were the same for each participant (within the same group), the spatial representation was different between participants. Moreover, mapping was re-randomized for each participant at the onset of each new daily training session in order to encourage generalizability.

Participants were randomly assigned to 1 of 3 groups. Group 1 received adaptive training using sequences with underlying structural regularities. Group 2 received adaptive training on sequences without underlying structural regularities. Group 3 received non-adaptive training on sequences without underlying structural regularities. For Groups 1 and 2, sequence lengths in the adaptive conditions were based on a 2 up 2 down staircase procedure. For example, if a participant started at sequence length 4 and correctly reproduced all items in that sequence then their next trial would be a sequence of length 4. If the participant correctly reproduced all elements in the second sequence of length 4 then they would move up to a sequence of length 5 in the next trial. If they incorrectly reproduced this sequence of length 5 then their next trial would be of a sequence of length 5. If they responded incorrectly to this sequence as well, then their next sequence would be moved down to length 4.

Whereas both Groups 1 and 2 incorporated the 2-up 2-down adaptive training staircase procedure, only Group 1 received sequences with structural regularities. Group 2 instead received adaptive training on sequences without any underlying structural regularities. In other words, this condition used the same 2 up 2 down staircase procedure as previously described; however, the sequences themselves were pseudo-randomly determined on each trial and were, therefore, without underlying structural regularities.

Finally, participants in Group 3 received training that did not adapt to their performance level. At each trial, the sequence lengths were determined randomly (varying in length from 4 to 16 elements). Like Group 2, the sequences in Group 3 were pseudo-randomly determined on each trial and, therefore, did not contain any underlying structural regularities.

#### Post-training measures

The post-training measures were identical to the pre-training measures. The post-training measures were administered in a separate session on the final day of the study, Day 6.

## Statistical Analysis

### Comparison of Group Means

A 3x2 mixed-design MANOVA with group (1, 2, 3) as the between-subjects factor and time of testing (pre, post) as the within-subjects factor was used to assess whether the training condition affected mean performance on the non-trained measure of SSP and the non-trained measure of language processing (both DVs described above). For the MANOVA, a given participant’s data was excluded if it was missing for either the pre or the post measure. This process resulted in data used in the analysis of both SSP and language processing for a total of N = 60. The MANOVA was performed using SPSS (IBM SPSS Statistics 21, Release Version 21.0.0.0).

### Mediation

In addition to the MANOVA, we used a mediational model analysis to estimate the relative direct and indirect effects of cognitive training. The IV was training type (Group), defined as the SSP training received by Group 1 (described above) and the WM training received by Group 2 (described above), both relative to the control, or reference, group (Group 3, described above). The DV was language processing (Language), defined as the correct number of degraded spoken target words for high-predictability sentences (H) minus the correct number of target words for anomalous sentences (A) at post-measure. The mediator (M) was structured sequence processing (SSP) defined as the span score for the grammatical sequences minus the span score for the ungrammatical sequences in the sequence learning task test phase at post-measure.

The process of dummy coding was used in order to enter the multicategorical, group-membership based IV (Group) into the model. Two dummy coded variables labeled D_1_ and D_2_ were created from observations in Group 1 and Group 2, respectively. The reference group was Group 3. No transformations were done on the continuous variables SSP and Language prior to entering them into the model. Variables were uncentered, and the data contained no outliers beyond +/- 3 standard deviations. Six subjects were excluded from the analysis due to missing data pertaining to the variables in the model. Thus, the final analysis included a total of N = 60 participants (Group 1 N = 21, Group 2 N = 19, Group 3 N = 20).

Mediation was based on an ordinary least squares regression approach [see [32] for a description] and was determined by a test of significance of the indirect effect of the IV on the DV through M, as this approach is preferred in contemporary mediation analyses [35], [36]. Because it is particularly appropriate for small sample sizes, we used the bootstrapping technique suggested by Preacher and Hayes [37], [38], [33] in which a point estimate of the indirect effect was obtained from the mean of 10,000 estimates of path *a x b* and 95% percentile-based confidence intervals were computed using the cut-offs for the 2.5% highest and lowest scores of the empirical sampling distribution, and adjusted for bias in the bootstrap distribution. The indirect effect was considered statistically significant if this bias-corrected confidence interval did not include 0.

All analyses of the mediation model were performed using SPSS (IBM SPSS Statistics 21, Release Version 21.0.0.0) with the Hayes and Preacher [33] macro MEDIATE that can be used for bootstrapped mediation models with a multicategorical variable.

## Results

### Comparison of Group Means

A 3x2 mixed-design MANOVA contrasting group (1, 2, 3) and time of testing (pre-post revealed a significant multivariate main effect on the linear composite of the DVs SSP and Language for time of testing (pre-post) [Wilks’ λ = .357, F(2, 56) = 50.534, p < .001, partial eta squared = .643] and no interaction with group [Wilks’ λ = .963, F(4, 112) = .539, p = .707, partial eta squared = .019].

Univariate follow-up analyses indicated there was no significant main effect for time of testing (pre-post) on SSP [F(1, 57) = .066, p = .799, partial eta squared = .001] and no interaction with group [F(2, 57) = 1.06, p = .353, partial eta squared = .036]. Looking at Table 3 at the mean values for the measure of SSP, only Group 1’s scores were higher from pre to post test. Both Group 2 and 3’s scores on the measure of SSP were worse from pre to post test. However, the univariate tests on the measure of SSP by itself indicated none of these changes were significant.

**Table 3:** Mean performance pre vs. post on measures of SSP and language processing.

	Group 1		Group 2		Group 3	
	M	S.D.	M	S.D.	M	S.D.
SSP (Pre)	19.71	16.18	20.74	16.75	16.00	19.75
SSP (Post)	26.81	21.54	18.47	17.42	13.55	21.19
Language (Pre)	3.52	2.04	4.68	2.63	4.20	2.19
Language (Post)	-0.86	2.24	0.32	2.50	0.05	2.21

There was a significant main effect for time of testing (pre-post) on Language, [F(1, 57) = 102.155, p < .001, partial eta squared = .642] and no interaction with group [F(2, 57) = .031, p = .969, partial eta squared = .001]. The univariate follow-up tests on Language by itself indicated all 3 groups showed significantly lower scores at the post-test on the measure of language, a finding that is further illustrated by the mean values in Table 3.

In summary, the MANOVA findings suggest overall lower language scores at post-training, but no effects or interactions that would suggest that the SSP training had significant effects on SSP or language. Therefore, Hypothesis 1 predicting that adaptive sequence training with structural regularities would result in improvements to both a non-trained SSP task and a language processing task, as compared to the other experimental group and the control group, was not supported.

### Mediation

**Fig 3:**
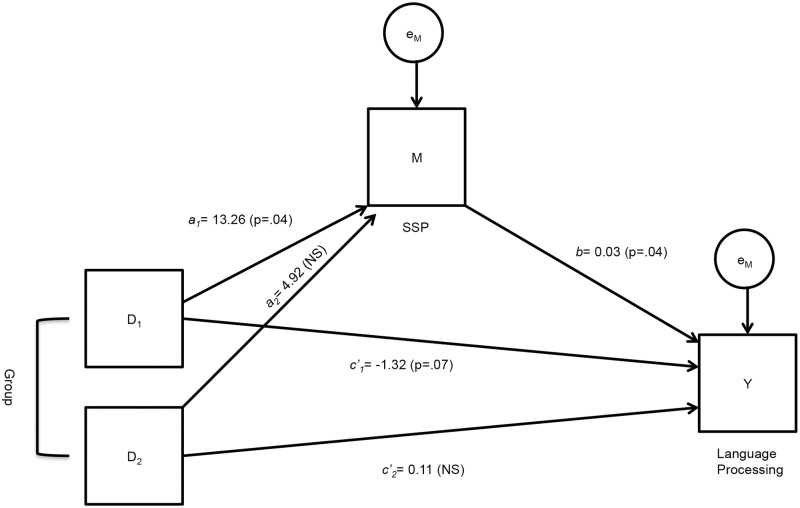
Final tested mediation model with unstandardized coefficients and significance values. Adaptive sequence training with structural regularities indirectly improved language processing through its enhancement of SSP (path *a*_1_ x *b*); whereas, adaptive sequence training with structural regularities directly worsened language processing (path *c'*_1_).

Although the group means did not demonstrate an effect of training, there is still utility in using the mediation analysis to determine whether there are underlying relationships among the constructs of interest. The final mediation model is presented in Fig 3. The results presented first pertain to the relative indirect effect of SSP training (Group 1) on Language, via SSP, compared to the control group and represented by path *a*_1_ x *b* of D_1_. From a mediation analysis conducted using bootstrapping, SSP training indirectly improved language processing through its enhancement of SSP. A 95% bias-corrected bootstrap confidence interval for the indirect effect (*a*_1_ x *b* = 0.42, SE = 0.29) based on 10,000 bootstrap samples did not include zero (0.03 to 1.25). What this shows is that participants in Group 1 who received adaptive sequence training with structural regularities were on average 13.26 units higher on SSP following training than participants in the control group, who received non-adaptive training on randomly-presented sequences (p = .04; see Table 4, path *a*_1_). Furthermore, holding type of training constant, those participants who were higher on SSP were also higher on Language (p = .03, path *b*). Given the relative indirect effect is a_1_ x *b*, participants in Group 1 who received adaptive sequence training with structural regularities performed 0.40 units better on Language as a result of the increase in SSP performance. In contrast, adaptive sequence training without structural regularities (Group 2) had no indirect effect on language processing. A 95% bias-corrected bootstrap confidence interval for the indirect effect (*a*_2_ x *b* = 0.15, SE = 0.21) based on 10,000 bootstrap samples did include zero (-0.17 to 0.71).

**Table 4:** Unstandardized coefficients.

	Unstandardized coeff	S.E.	t	p
Path a_1_ indirect	13.26	6.31	2.10	.04
Path b indirect	0.03	.01	2.13	.04
Path c'_1_ direct	-1.32	.73	-1.81	.07
Path c_1_ total	-.91	.72	-1.25	.22
Path a_2_ indirect	4.92	6.47	.76	.45
Path c'_2_ direct	.11	.72	.15	.88
Path c_2_ total	.27	.74	.36	.72

Note. R^2^ = 0.12, Adj. R^2^ = 0.07; F(3,56) = 2.52, p = .07 (DV Model, Outcome Variable: Language)

The results presented next pertain to the relative direct effect of SSP training (Group 1) on Language, compared with the control group and represented by path *c'*_1_ (see Fig 3). The results of the analysis revealed that SSP training directly lowered language processing. Specifically, when considering the direct path in isolation, participants who received adaptive sequence training with structural regularities were on average 1.32 units worse on Language than participants who received non-adaptive, randomly-presented sequences (p = .07; see Table 4, path *c'*_1_). Thus, Hypothesis #3, predicting that adaptive sequence training with structural regularities would have a positive effect on language processing, was not supported. Furthermore, the direct effect of WM training (Group 2) on Language, compared with the control group, was not significant (*c'*_2 =_ .11, p = .88; see Table 4).

According to the approach implemented by Zhao, Lynch, and Chen [36], since the product of *a*_1_ x *b* x *c'*_1_ is negative (-0.53), this indicates that the model corresponding to SSP training (Group 1) is one of competitive mediation. These authors point out that if the direct effect *c'* is substantially larger than the indirect effect *a* x *b*, then the total effect *c* would also be negative [given the formula for the total effect is *c* = (*a* x *b*)+ *c'*)] [36]. Potential implications of this unpredicted finding will be addressed in the Discussion.

## Discussion

The results of the comparison of group means (using MANOVA) did not support our first hypothesis. That is, adaptive sequence training with structural regularities did not lead to a significant improvement to SSP or language processing. There are a number of reasons why a non-significant result might have been obtained. For instance, it is possible that the training regimen was not strong or consistent enough to lead to significant improvements overall, even if the training itself has the potential to have a causal effect on SSP and language. Thus, even though the MANOVA resulted in non-significant effects of SSP training, using the mediation model allows us to better understand the potential underlying relationships among the variables of interest.

Along these lines, the results of the mediation model did show that there was a significant relative indirect effect of adaptive sequence training with structural regularities on language via SSP, compared with the control condition, supporting Hypothesis #2. This relative indirect path shows that SSP training has an underlying impact on language––specifically, the ability to use knowledge of word order statistics to improve speech recognition in noise––through the mediator SSP. Previous research using an individual differences approach has established an empirical association between SSP and language ability [21], [5], [20]. Furthermore, there is evidence that language impairments are associated with impairments to SSP, e.g., [17]. However, the present findings are the first results that we know of that demonstrate a more direct connection between SSP and an aspect of language processing as revealed through experimental manipulation of SSP training itself. Likewise, these are also the first findings demonstrating that cognitive training techniques can potentially modify SSP.

The mediation model demonstrates that adaptive sequence training with structural regularities has the potential to generalize to improvements on a non-trained statistical-sequential learning task, as shown in path *a*_1_. This finding itself has remarkable implications about the plasticity of SSP and statistical learning processes and the potential for using such sequence training tasks to improve fundamental learning (and language) abilities. Thus, even though an examination of the group means revealed no significant effects of SSP training, the mediation model suggests that underlying relationships among these variables exist, providing the basis to further develop such training techniques to improve SSP and language.

In terms of Hypothesis #3, the significant indirect effect of *a*_1_ x *b* was accompanied by a significant––and opposite, or competing––direct effect (path *c'*_1_). Thus, Hypothesis #3 was not supported. According Zhao, Lynch, and Chen [36], our model is one of competitive mediation, in which the total effect *c*_1_ is negative (-0,91, p = .22, see Table 4), [given the formula for the total effect is *c* = (*a* x *b*)+ *c'*)] [36]. It is possible that there is an unexplained mediator(s) in the direct path, thereby contributing to a negative relationship between SSP training and language. Although this is a valid possibility, it is also possible that SSP training directly negatively impacts language (through some as yet unexplained mechanism), and is thereby responsible for the negative total effect *c*_1_. This second possibility would not require an additional mediator, only an additional explanatory construct to explain how the variables already in the model behave on each of the different paths.

Why does SSP training have a positive underlying relationship with language performance via the indirect effect through the mediator SSP, but overall a negative underlying relationship with language performance via the direct effect? One intriguing possibility is that training on visual structural regularities––apart from any effect it has on SSP––might actually interfere with one’s knowledge or use of the structural regularities in spoken language. Recall that the measure of language was derived from a difference score: performance with high-predictability sentences (containing language regularities) minus performance with anomalous sentences (containing fewer regularities). An examination of the means on the high-predictability and anomalous sentences suggests that only in Group 1 does performance on the highly-predictable sentences get substantially worse from pre- to post-training. Performance on the anomalous sentences on the other hand does not worsen for any of the three groups. Thus, knowledge (or expression of) language regularities as measured by the highly-predictable sentences appears to be attenuated following sequence training with structural regularities. A similar type of interference was recently observed when participants engaged in a non-linguistic visual-motor SSP task concurrent with a sentence comprehension task [39]. This interference between the processing of visual structural regularities (contained in the sequence training task) and processing spoken language regularities (contained in the highly-predictable sentences) could be the mechanism driving the negative direct effect of path *c'*_1_. With these two competing pathways––an indirect benefit to language processing by strengthening SSP, but a simultaneous direct negative effect on language due to interference––the final effect in our case is an overall negative total effect or decrease in language processing. Theoretically, this is quite enlightening, and furthermore underscores the complex relationship between SSP and the processing of language regularities and sheds light upon why in some cases a near transfer effect but not a far transfer effect might be observed. In this case, the near transfer effect (improvement to SSP) would normally result in a far transfer effect (improvement to language), but because the training regimen itself has a negative direct effect on language, it cancels out the potential beneficial improvement to language.

As the above discussion helps illustrate, these findings accentuate the importance of pursuing designs that allow one to better understand the mechanisms underlying training-related changes to outcome measures. Some recent literature has been more critical of computerized WM training studies and has brought into question the generalizability of the findings e.g., [31]. The advantage of our design is that it helps inform the process of change, rather than simply comparing means of the changes to the outcomes themselves; we believe this may help address the concerns pertaining to the effectiveness of cognitive training techniques and possibly provide answers as to why near and far transfer effects are observed. In turn, this can serve to maximize the potential of these interventions. With a growing number of reports claiming the promise of cognitive training techniques, as well as other more recent studies that are more critical of their validity, our findings might offer clarity into the mechanisms of change themselves. For instance, given the present results suggesting the existence of two independent pathways for how sequence training might affect language, it is possible that previous training studies have used protocols that inadvertently emphasize one or the other pathway, leading to very different and inconsistent results across studies. In fact, a given experimental manipulation, such as changing the sensory modality or duration of the training task, may have different effects on the strength of each pathway. Discovering what the key manipulations are and how each affects different mechanisms of change is an important next step. With such knowledge, it may be possible to design a training regimen that better capitalizes on the indirect path in order to yield the greatest chance of demonstrating far transfer, in this case, enhancement to language performance. Importantly, the existence of these two competing paths would not have been known had one relied solely on the MANOVA analyses comparing the group means.

Likewise, it is possible that there may be ways to modify the training task to alleviate the negative, direct path between training and language performance. For instance, having a longer training regimen could provide an opportunity for participants to adapt to the novel structural regularities that they are exposed to, reducing the interference between the training task and language processing. If this is true, then with additional training trials, the direct (negative) effect of adaptive training with structural regularities on language might decrease, resulting in even stronger enhancements to language. Another possibility might be to modify the type of structural regularities that are present in the training sequences. For example, rather than having only adjacent sequential dependencies as was the case here, incorporating long-distance, non-adjacent dependencies into the training task might prove beneficial for improving the mechanisms that are recruited when processing long-distance dependencies in natural language [40],[41].

Although the positive underlying relationship between SSP and language (illustrated in path *b* shown in Fig 3), may seem relatively small (*b* = .03), it is still encouraging. It is true that the overall indirect path *a*_1_ x *b* may at first suggest that it takes a substantial improvement to SSP to see a small improvement to language processing; however, a few considerations are important to mention. First, the present design incorporated only 4 training sessions, a small number. It seems highly possible that with more training sessions, or by modifying the training task in some other way, even greater enhancement to SSP might be obtained, which could in turn boost the language score even more. Second, considering the long-term applied goal of how these types of cognitive training regimens might be used to improve language in typical and atypical development, even a small gain could have a major impact, especially if such an intervention was targeted early in development.

## Conclusions

In conclusion, using a mediation model analysis, we have demonstrated that adaptive sequence training with structural regularities has an underlying impact on language through the mediator SSP. More concretely, adaptive sequence training with structural regularities has a very large impact on SSP while changes to SSP, in turn, have a more modest impact on language processing. In competition with this indirect effect, our sequence training regimen appeared to have a negative direct effect on language performance as well, resulting in overall a more dampened effect on language ability and no overall improvements to language as assessed by MANOVA. Additional work is currently underway to understand this negative direct effect, with the aim of reducing its impact to improve the efficacy of such sequence training programs.

These findings have two implications. At a practical level, these findings show how fundamental learning abilities and language processing skills might be improved in typical and atypical development. At a theoretical level, these findings not only highlight the plasticity of statistical-sequential learning and SSP but they also show an underlying link between SSP and language, which in turn lends additional weight to the view that language acquisition is based in large part on domain-general learning mechanisms rather than language-specific modules or neural structures that solely mediate language alone.
